# Urethral Stricture

**Published:** 1885-10

**Authors:** Herman E. Hayd

**Affiliations:** M. D., M. R. C. S., Eng.; No 9 Niagara Street


### An Interesting Case of Urethral Stricture, Complicated with Scrotal and Perineal Fistulæ.

By Herman E. Hayd, M. D., M. R. C. S., Eng.

W. S., married, aged 37, came under my care March 9th, with the following history :

About fourteen years ago, contracted gonorrhœa, which lasted some sixteen months. Three years later, got what he called a second dose which only lasted a few days. Since that time, had often noticed a slight discharge after any excessive sexual indulgence. In 1876, had a chancre, which was followed by the ordinary secondary manifestations. For the past thirteen months, he had complained of frequency of micturition—a difficulty and straining during the act—and attended with much pain. These symptoms became more and more troublesome, until retention of urine took place, which was often relieved by rest, hot fomentations, sitz baths, etc., etc. But upon one occasion these measures did not relieve him ; he suffered terrible cramps, a feeling of great distension in the abdomen, much pain and tenderness over the pubic region, weight and bearing down in the perineum, frequent and painful urination. In the course of a few days, a large swelling appeared in the lower portion of the right scrotum. This was poulticed, and finally broke, and urine escaped freely through the opening during micturition. A second swelling appeared in the right perineum a few weeks later. It was also poulticed, and in its turn broke, and thus another opening was established, through which urine flowed. This condition remained for some weeks, when he decided to come to Buffalo.

I saw the patient for the first time on the morning of the 9th of March, after having traveled by rail nearly one hundred and fifty miles, and, to say the least, found him in a most pitiable condition.

He was pale and emaciated, cheeks flushed, lying on his back with legs flexed and knees elevated, and suffering intense agony.

Large rupial crusts were seen upon the body, especially upon the extensor surfaces of the extremities. The left alæ nasi was much swollen, and deeply ulcerated, with irregular cicatrices leading to it. The scrotal and perineal openings were closed, and the perineum itself greatly swollen, hard, tense and excessively tender and painful. The anal orifice was pushed downwards and backwards, its edges gaping, and the sphincter ani much relaxed. The right half of the scrotum enlarged and swollen, and filled with fluid, and bound down to the adjacent tissues. The skin of a deep, bluish red color, testicle enlarged, and spermatic cord thickened and tender. Tongue dry and coated. Temperature, 103 2-5 ; pulse, 120, soft and compressible.

I at once made an incision through the closed scrotal fistula, and a half pint of dirty, stinking fluid, with a strong, urinous smell escaped. Patient was then asked to make water, and by getting on his elbows and knees—a position very familiar to him—he succeeded in passing through this opening, after considerable effort, quite a lot of a stinking, ammoniacal urine, none escaping through the meatus. The scrotum was then gently washed out with a weak solution of carbolic acid in warm water, a drainage tube inserted into the wound, hot poultices of linseed meal, with tincture opium, applied to the perineum and scrotum, and the following prescription ordered: Quiniæ sulph. in capsule grs. iv. every third hour; pil. morph. sulph. grs. ¼ every second hour, and an abundance of rich broths, and eggs beaten up with a very little whiskey.

March 10. Patient had a very restless night, suffering much pain in the perineum, had repeated chills and high fever; urinated every ten or fifteen minutes, and suffered intensely. Upon examination, felt deep fluctuation in perineum. An incision was made on each side of the median raphe, and pus flowed freely from the part. Carbolized water was again injected through the scrotal opening, and escaped freely through both perineal incisions, the communicating canal being five inches in length. Temperature, 102 2-5 ; pulse, 115. Ordered abscess to be syringed night and morning. Continued quinia and morphia, but to be given less frequently, and ordered a saline diuretic mixture; Potassæ acetatis 1 dr., tinct. hyosy. 1 dr., every third hour.

March 11. Patient had a better night, slept very well, complained of some pain in perineum; made water every second hour, accompanied with great burning and scalding. The principal portion of the urine passed through the scrotal opening, although, when the flow had attained its greatest force, urine passed freely through both perineal incisions. Palpation over bladder revealed some tenderness, and the percussion was dull two inches and a half above pubes. Temperature 99 1-5; pulse, 100; tongue moister and appetite returning.

Chloroform was administered to the patient, and an examination of the urethra carefully made with a No. 10 flexible French bougie. About four and a half inches from meatus urinaris, an obstruction was felt, through which a small filiform bougie—after careful and patient manipulation—was passed, and over it an Otis dilator, which was forced through the stricture, and rapidly and forcibly expanded to No. 35 on scale-register. An attempt was then made to pass a small-sized bougie into the bladder, but this was impossible, as the urethra behind the stricture was widely dilated, honey-combed and riddled with irregular passages caused by the dammed-up urine. All available expedients were tried. The urethral canal was injected full of olive oil, and the fistulous openings held shut by an assistant, when an attempt was made to pass a small steel sound with a long prostatic curve into the bladder; then one with a short, abrupt bend was tried, and finally a number of filiform bougies, one along the side of the other, but without success.

Before deciding upon anything so serious as external urethrotomy, I resolved to wait to see what the morrow might bring forth, feeling satisfied that little harm could result, as the anterior stricture was already broken down — undoubtedly the cause of the urinary extravasation — since it took place in front of the triangular ligament, and had worked itself forward into the scrotal tissues.

March 12. Patient is quite comfortable; complains of some pain and tenderness in perineum. Had no chills or fever last night. Made water very frequently; stream small, but flows quite freely through meatus urinaris. Scrotal sinus discharging, very freely, a thick, stringy, lumpy, muco-purulent matter. Temperature, 99; pulse, 90. Tongue moist and clean. Continued the quinia and morphia, and ordered the following:

Pot. Iod., - - - - - grs. vii
Tinct. Cinchon. Co., - - - - m. xxx
Spts. Ether Chlor. M. V., ter. in. die sumend.

The patient's condition continued to improve; the sinuses were dressed night and morning, and in a few days he sat up. His chief complaint now was a difficulty in making water, not from any great pain during the act, but the time and effort required to accomplish it. There was also a slight degree of tenderness over the pubic region.

March 17. Patient was again put under chloroform, and a No. 18 English blunt-pointed bougie was passed, until an obstruction was met in membraneous urethra, six and a quarter inches.

I now attempted to pass a No. 8 American metallic catheter into the bladder, and succeeded in doing so, but the instrument was firmly grasped by a stricture in the membraneous urethra.

Permit me here to say, that I had specially prepared the point of this instrument for another case, where I operated with the greatest satisfaction. I simply filed off the under surface of the point, so that if I succeeded in getting it into the bladder, I might pass a long, thin filiform bougie through it, then withdraw the catheter, and use the bougie already in the bladder as a guide for the dilator.

This I subsequently did, and dilated the posterior stricture with a Gouley dilator sufficient to enable me to pass at once a

No. 18 English sound into the bladder. Upon several occasions, I have felt highly gratified while using this same catheter. Previous to my taking advantage of this idea, I did, no doubt, what many of my readers have often done. After a great amount of patience and careful manipulation, have succeeded in passing a bougie or sound through a urethra riddled with false passages, and then, after withdrawing that size, with the hope of getting in a larger one, could not find the once-found passage, or only after the greatest trouble, and after having inflicted much injury and unnecessary irritation and subsequent pain and distress to the patient.

During the past six months, I have forcibly dilated seven strictures with a Gouley and Schweig's dilator. In four cases, the stricture existed in the spongy urethra, and, in the other three, two strictures were present, one in the spongy portion, and a posterior one in the membraneous urethra, at the bulb. So far, my experience of the treatment of urethral stricture by divulsion has been highly satisfactory, having effected a cure in every case, and in none have I had any complications follow. These cases occurred in private practice, and have consequently been under my observation from time to time, and every few weeks I have passed a large steel sound, so that no subsequent contraction could take place.

March 18. Patient very comfortable and cheerful; slept better, and had fewer calls to make water. An hour after the operation of yesterday, the patient passed nearly a pint of water, the stream flowing freely through both perineal fistulæ, scrotal fistula and meatus urinaris. The sinuses were again syringed and washed with carbolic acid water, and dressed, and the patient allowed to sit up.

Day by day, marked improvement took place, and on the fourteenth day the perineal fistulæ were closed, and on the twenty-first day the swelling of the scrotum and perineum had all disappeared, and the scrotal sinus nearly healed up.

Emol. Ol. Morrhuæ was ordered ℥ss ter. in. die, and the patient directed to go out for a short walk.

I passed a No. 14, 16 and 18 steel sound every week at one sitting, and on the ninth week the patient was let go home, after having been carefully instructed how to pass the instruments himself. Before leaving Buffalo, the patient had gained much in flesh, rupial crusts had nearly all disappeared, and the ulceration of the left alæ nasi had practically ceased, and the tissues were healing kindly. He continued both mixtures, the Iodide of Potash and the Cod Liver Oil emulsion, for three months, and on June 3d called again to see me, when I passed a No. 18 English sound. The fistulæ were all closed, and the patient in good health and spirits. He was advised to pass the sounds once a month for a few months, then every two months, and gradually increase the intervals.

It is interesting to note what a marked and rapid improvement took place in the patient in so short a time, and how quickly the syphilitic condition responded to the small doses of Iodide of Potash and the Cod Liver Oil. Indeed, I have sometimes thought that Cod Liver Oil, in the late stages of syphilis, seems to act as a specific. Repeatedly, I have had patients under my care, who had been furiously dosed with mercury and large and increasing doses of Iodide of Potash, without any apparent good, rapidly get well under a tonic course of Elix. Quiniæ Ferri. et Strych. and an Emul. of Ol. Morrhuæ.

No 9 Niagara Street.

